# Clinical Outcomes of COVID-19 Convalescent Plasma According to Treatment Timing and ELISA Antibody Levels: A Real-World Retrospective Cohort Study

**DOI:** 10.3390/medicina62091649

**Published:** 2026-08-28

**Authors:** Özlem Bayraktar Saral, Ayla Yavuz, Şenol Ardıç, Tuncay Yazıcı, Serap Özer Yaman

**Affiliations:** 1 Department of Infectious Diseases and Clinical Microbiology, Faculty of Medicine, Trabzon Kanuni Training and Research Hospital, Trabzon University, Trabzon 61335, Türkiye; 2 Blood Center, Trabzon Kanuni Training and Research Hospital, Trabzon University, Trabzon 61335, Türkiye; 3 Department of Emergency Medicine, Faculty of Medicine, Trabzon Kanuni Training and Research Hospital, Trabzon University, Trabzon 61335, Türkiye; 4 Department of Emergency Medicine, Bayburt State Hospital, Bayburt 69000, Türkiye; 5 Department of Medical Biochemistry, Faculty of Medicine, Trabzon Kanuni Training and Research Hospital, Trabzon University, Trabzon 61335, Türkiye

**Keywords:** COVID-19, convalescent plasma, passive immunotherapy, ELISA index, antibody level, treatment timing, mortality

## Abstract

*Background and Objectives*: Convalescent plasma (CP) was widely used during the early phase of the COVID-19 pandemic; however, evidence regarding its clinical benefit has remained inconsistent. This study evaluated the associations of CP administration timing and the ELISA antibody level of the transfused unit with clinical outcomes among patients with COVID-19. *Materials and Methods*: This retrospective real-world cohort study included 272 adults with RT-PCR-confirmed COVID-19 who received a single CP unit at the University of Health Sciences Trabzon Kanuni Training and Research Hospital, Türkiye, between 1 April 2020 and 1 April 2021. At CP administration, 180 patients were hospitalized, and 92 were treated as outpatients; initial cohort assignment was retained throughout follow-up. Anti-SARS-CoV-2 IgG was assessed using the semi-quantitative Euroimmun ELISA, and exploratory low (0.00–0.99), medium (1.00–4.99), and high (≥5.00) categories were used. The primary outcome was 30-day all-cause mortality. *Results*: During follow-up, 18 of 92 outpatients (19.6%) required hospitalization. Thirty-day mortality was higher among hospitalized patients than among outpatients (39.4% vs. 1.1%; *p* < 0.001), reflecting major baseline clinical differences between the groups. Median ELISA indices did not differ significantly between hospitalized patients and outpatients [4.23 (IQR, 2.65–6.43) vs. 3.64 (IQR, 2.16–6.13); *p* = 0.308]. Among hospitalized patients, ELISA-index distributions were similar between survivors and non-survivors (*p* = 0.427), and the index was not associated with mortality when analyzed continuously after log2 transformation (OR per doubling, 0.89; 95% CI, 0.67–1.19; *p* = 0.425). The high-versus-medium category comparison was also non-significant (OR, 0.88; 95% CI, 0.48–1.62; *p* = 0.757), as was the timing-by-ELISA interaction (*p* = 0.074). *Conclusions*: In this CP-treated cohort, the ELISA index of the transfused unit was not independently associated with 30-day mortality. The exploratory subgroup findings do not establish CP effectiveness or superiority of a particular timing or antibody-level category.

## 1. Introduction

Coronavirus disease 2019 (COVID-19), caused by severe acute respiratory syndrome coronavirus 2 (SARS-CoV-2), was first identified in Wuhan, China, in December 2019 and rapidly evolved into a global pandemic, posing an unprecedented challenge to public health worldwide [1,2]. Although the clinical spectrum of COVID-19 ranges from asymptomatic infection to mild upper respiratory tract illness, severe pneumonia, acute respiratory distress syndrome (ARDS), multiorgan failure, and death may occur, particularly in older adults and patients with underlying comorbidities or immunosuppressive conditions [1,2].

The progression of severe COVID-19 is characterized by high viral replication during the early phase of infection, followed by dysregulated immune responses, endothelial injury, and excessive systemic inflammation. These mechanisms contribute to cytokine release, ARDS, thromboembolic complications, multiorgan dysfunction, and increased mortality [3]. Elevated inflammatory biomarkers, including C-reactive protein (CRP), ferritin, D-dimer, and profound lymphopenia, have consistently been associated with poor clinical outcomes [1,2,3]. Consequently, therapeutic strategies capable of suppressing viral replication during the early stage of infection became a major focus during the initial phase of the pandemic.

Before effective antiviral agents and vaccines became widely available, passive immunotherapy emerged as one of the most promising treatment approaches. Convalescent plasma (CP) therapy involves transfusion of plasma obtained from individuals who have recovered from SARS-CoV-2 infection and contains virus-specific antibodies. Although viral neutralization is considered a principal mechanism of action, CP may also modulate complement activation, enhance antibody-mediated immune responses, and attenuate excessive inflammation. Previous experience with SARS, Middle East respiratory syndrome (MERS), influenza, and Ebola further supported the potential use of CP during the early stages of the COVID-19 pandemic [4,5,6].

Evidence regarding the clinical efficacy of CP has remained inconsistent. Several observational studies suggested that early administration of high-antibody-level CP reduced mortality, accelerated clinical recovery, and decreased hospitalization rates, particularly when administered during the viral replication phase [7,8,9]. In contrast, large randomized controlled trials, including the RECOVERY trial and subsequent systematic reviews and meta-analyses, failed to demonstrate a consistent survival benefit across all patient populations [10,11,12,13,14]. These apparently conflicting findings may partly reflect substantial heterogeneity in patient selection, baseline disease severity, timing of CP administration, recipient serostatus, antibody levels in transfused plasma, laboratory assays used to characterize antibody content, and concomitant therapies [9,10,11,12,13,14].

Real-world studies may complement randomized controlled trials by providing additional evidence on the clinical outcomes of CP therapy in routine clinical practice. However, relatively few real-world studies have simultaneously evaluated the timing of CP administration and antibody levels in transfused plasma. Furthermore, the antibody levels that may be associated with more favorable clinical outcomes and the patient populations most likely to benefit remain uncertain [9,12,13,14]. Differences in antibody assays and treatment protocols further complicate comparisons across studies. Therefore, a better understanding of the relationship between CP administration timing, antibody levels, and clinical outcomes remains important for interpreting the COVID-19 experience and may also inform the potential use of passive immunotherapy in future emerging viral outbreaks.

The present real-world retrospective cohort study aimed to evaluate the associations of antibody levels in transfused convalescent plasma and the timing of CP administration with clinical outcomes, including 30-day all-cause mortality and, according to the initial treatment setting, namely ongoing ICU care or discharge by day 30 among hospitalized patients and subsequent hospitalization among patients initially treated as outpatients with COVID-19 during the pre-vaccination period.

## 2. Materials and Methods

### 2.1. Study Design and Patient Population

This retrospective observational cohort study was conducted at the University of Health Sciences Trabzon Kanuni Training and Research Hospital, Türkiye. Adult patients (≥18 years) with laboratory-confirmed COVID-19 who received convalescent plasma (CP) therapy between 1 April 2020 and 1 April 2021 were included. COVID-19 was confirmed by real-time reverse transcription polymerase chain reaction (RT-PCR) performed on nasopharyngeal swab specimens. Pregnant patients were excluded.

A total of 272 patients were included in the study. At the time of CP administration, 180 patients were hospitalized and 92 received CP therapy as outpatients. Patients were classified as hospitalized patients or outpatients according to their treatment setting at the time of CP administration, and their initial group assignment was maintained throughout follow-up. Of the 92 patients who received CP as outpatients, 18 subsequently required hospitalization because of clinical deterioration; however, these patients remained in the outpatient cohort, and subsequent hospitalization was evaluated as a secondary clinical outcome.

Only patients who received a single CP transfusion and for whom the antibody level of the transfused plasma unit was available were included in the analysis.

**Figure 1:**
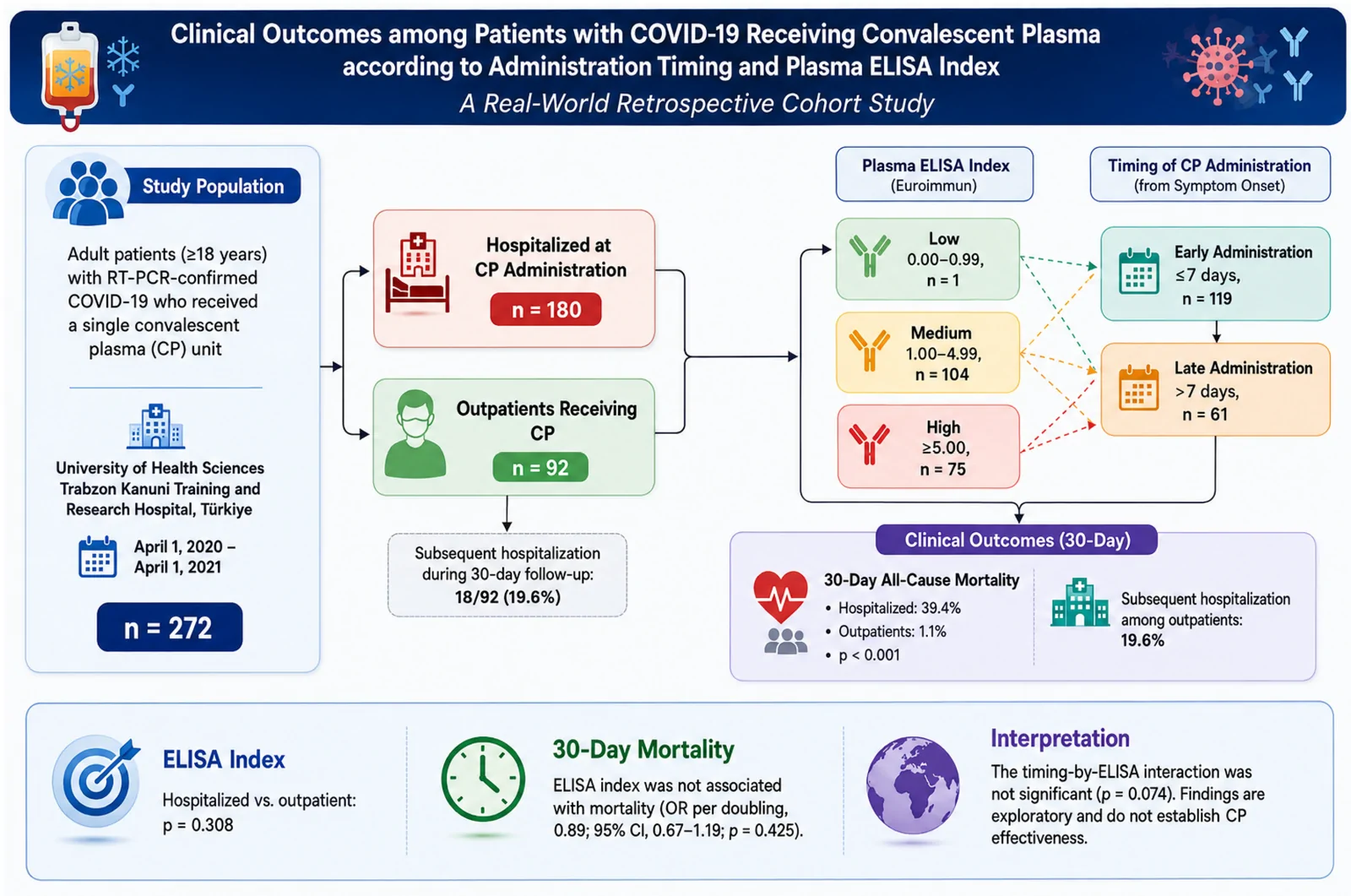
Graphical overview of the study design, patient flow, ELISA antibody-level classification, treatment timing, and clinical outcome assessment.

A graphical overview of the study design and analytical workflow is presented in Figure 1. Patients were enrolled according to the predefined eligibility criteria and classified as hospitalized patients or outpatients based on their treatment setting at the time of CP administration. Patients were subsequently evaluated according to the timing of CP administration and antibody levels in the transfused plasma. The predefined outcomes were 30-day all-cause mortality for the full cohort; mutually exclusive 30-day clinical-status categories of death, ongoing ICU care, or discharge among hospitalized patients; and subsequent hospitalization during follow-up among the cohort initially treated as outpatients.

### 2.2. Convalescent Plasma Administration

Convalescent plasma therapy was administered in accordance with the Republic of Türkiye Ministry of Health recommendations for the management of adult patients with COVID-19 and the National Guideline for the Procurement and Clinical Use of COVID-19 Immune (Convalescent) Plasma that was in effect during the study period.

The decision to manage patients as outpatients or inpatients was based on the disease severity and hospitalization criteria defined in the national COVID-19 guideline in effect during the study period. Hospitalization was considered in patients with clinical findings indicating more severe disease or an increased risk of deterioration, including respiratory distress, increased respiratory rate, hypoxemia, pulmonary involvement on imaging, and poor prognostic laboratory findings. Patients who met hospitalization criteria or showed clinical deterioration were managed as inpatients. In contrast, patients who did not meet hospitalization criteria and were clinically stable at the time of CP administration were treated as outpatients. Outpatients receiving CP were nevertheless considered at increased risk of disease progression because of advanced age, immunosuppression, underlying comorbidities, or other clinical risk factors. Clinical assessment included vital signs, oxygen saturation, and pulmonary imaging findings, as clinically indicated.

During the study period, some hospitalized patients received more than one CP unit according to clinical need. However, because antibody levels could differ between individual plasma units, a single and clearly defined antibody exposure could not be assigned to patients who received multiple CP units. Since the primary objective of the present study was to evaluate the association between the antibody level of the transfused plasma and clinical outcomes, only patients who received a single CP unit were included in the present analysis. Accordingly, antibody exposure for each included patient was defined by the ELISA index value of the single transfused plasma unit.

All plasma units were collected from recovered COVID-19 donors and supplied by the Turkish Red Crescent in accordance with the national guideline for convalescent plasma procurement and clinical use. The CP units supplied by the Turkish Red Crescent had a standard volume of 200 mL. Before CP transfusion, ABO blood group compatibility was confirmed, and patients were evaluated for IgA deficiency. Each CP unit was administered over approximately 90–120 min under close clinical observation. No life-threatening transfusion-related adverse events were observed during the study period.

### 2.3. Measurement of Antibody Levels in Convalescent Plasma

Anti-SARS-CoV-2 IgG antibody levels in CP units were assessed using the semi-quantitative Anti-SARS-CoV-2 ELISA IgG assay (Euroimmun, Lübeck, Germany), which targets the S1 domain of the SARS-CoV-2 spike protein. The assay output was expressed as a unitless ELISA index (sample-to-calibrator ratio) and was obtained for each transfused unit from the Turkish Red Crescent database.

For each included patient, antibody exposure was defined as the ELISA index of the single CP unit transfused. The ELISA result was available in the blood-product record before transfusion; however, plasma-unit allocation was based on ABO compatibility and product availability rather than a prespecified patient-level ELISA-index target.

For exploratory analyses, ELISA indices were categorized post hoc into low (0.00–0.99), medium (1.00–4.99), and high (≥5.00) antibody-level groups using pragmatic round-number cutoffs on the semi-quantitative ELISA-index scale. These thresholds were used to generate ordered exposure strata and were not derived from receiver operating characteristic analysis, outcome-based optimization, manufacturer-defined diagnostic categories, neutralizing-antibody measurements, or validated clinical efficacy thresholds. Because only one hospitalized patient was classified in the low-level group, estimates for this category were considered statistically unstable. Therefore, categorical analyses were regarded as exploratory and were supplemented by regression analyses treating the ELISA index as a continuous variable. Owing to its right-skewed distribution, the index was log2-transformed, and the corresponding odds ratios were interpreted per twofold increase in the ELISA index.

### 2.4. Study Outcomes

The primary analytical population comprised all eligible patients who received one CP unit. The primary exposure was the ELISA index of the transfused unit, and the primary outcome was 30-day all-cause mortality, with time zero defined as the date of CP administration.

Thirty-day vital status was ascertained using the national electronic health record system (e-Nabız) and integrated public healthcare information systems. These interconnected electronic systems enable authorized healthcare professionals to access health information recorded across public healthcare institutions at the national and regional levels. Therefore, follow-up was not restricted to records from the study hospital, and post-discharge vital status could also be assessed for patients discharged before day 30. Thirty-day vital status was available for all patients included in the analysis, and no patients were lost to follow-up for the primary 30-day mortality outcome.

Secondary outcomes were assessed during the 30 days after CP administration. Among patients treated initially as outpatients (*n* = 92), subsequent hospitalization during the 30-day follow-up was evaluated as a post-treatment outcome while initial cohort assignment was retained. Among patients who were hospitalized at the time of CP administration (*n* = 180), the recorded 30-day clinical status was classified into mutually exclusive categories of death, ongoing ICU care at day 30, or discharge by day 30. The analytical population and denominator for each secondary outcome are reported with the corresponding analysis.

Treatment timing was defined as the interval from patient-reported symptom onset to CP administration. Patients were categorized as early (≤7 days) or late (>7 days). The cutoff was prespecified on the basis of the biological rationale for passive antibody administration during the viral-replication phase and the approach used in earlier clinical studies, including a previous outpatient cohort from the same center. Because symptom onset was obtained retrospectively, recall-related misclassification was possible. The interval was also analyzed continuously when available.

Subgroup analyses compared early (≤7 days) versus late (>7 days) CP administration and evaluated 30-day clinical outcomes according to antibody levels in the transfused plasma. Among hospitalized patients, exploratory analyses evaluated the associations of treatment timing and the study-specific antibody-level categories with 30-day mortality and ongoing ICU care at day 30. In addition to the study-specific antibody-level categories, the ELISA index was also evaluated as a continuous variable.

### 2.5. Ethical Approval

The study protocol was approved by the Non-Interventional Clinical Research Ethics Committee of the University of Health Sciences Trabzon Kanuni Training and Research Hospital (Decision No. 2021/10; 10 June 2021). The ethics committee waived study-specific informed consent for this retrospective analysis. Written informed consent for CP transfusion had been obtained from all patients as part of routine clinical care.

### 2.6. Statistical Analysis

Statistical analyses were performed using IBM SPSS Statistics for Windows, version 23.0 (IBM Corp., Armonk, NY, USA). Continuous variables were assessed for distributional characteristics and are presented as mean ± SD or median (IQR), as appropriate. Between-group comparisons used Welch’s t test or the Mann–Whitney U test for continuous variables and the chi-square or Fisher’s exact test for categorical variables; Fisher’s exact test was preferred when expected cell counts were <5. Effect estimates are reported as odds ratios (ORs) with 95% confidence intervals (CIs) where estimable. The ELISA index and symptom-onset-to-CP-administration interval were additionally analyzed as continuous variables. Because the ELISA index and CRP were right-skewed, they were log2-transformed for regression. An exploratory multivariable logistic regression model for 30-day mortality among hospitalized patients included age, sex, comorbidity status, baseline oxygen-support category, log2-transformed CRP, treatment interval, and log2-transformed ELISA index. A timing-by-ELISA interaction term was evaluated in a separate exploratory model. Complete-case analysis was used; no values were imputed. Two-sided *p* < 0.05 was considered statistically significant, and subgroup and interaction analyses were regarded as exploratory.

## 3. Results

### 3.1. Patient Characteristics

A total of 272 adult patients with RT-PCR-confirmed COVID-19 who received a single convalescent plasma (CP) unit were included in the study. At the time of CP administration, 180 patients were hospitalized, whereas 92 received CP as outpatients. Patients were classified as hospitalized patients or outpatients according to their treatment setting at the time of CP administration, and this baseline group assignment was maintained throughout follow-up.

Of the 92 patients who initially received CP as outpatients, 18 (19.6%) subsequently required hospitalization because of clinical deterioration. These patients were not reclassified into the hospitalized cohort; instead, subsequent hospitalization was considered a secondary clinical outcome within the outpatient cohort.

No life-threatening CP transfusion-related adverse events were observed during the study period.

Demographic and clinical characteristics of the study population are summarized in Table 1. The mean age was 68.6 ± 13.8 years in hospitalized patients and 66.3 ± 9.5 years in outpatients. Women accounted for 50.6% (91/180) of the hospitalized group and 52.2% (48/92) of the outpatient group. The distribution of comorbidities and CP-related characteristics according to treatment setting is also presented in Table 1.

The median time from symptom onset to CP administration was 5.5 days (IQR, 3–8) in hospitalized patients and 4 days (IQR, 4–6) in outpatients. Early CP administration (≤7 days) occurred in 119/180 (66.1%) hospitalized patients and in all 92 (100%) outpatients. The median ELISA index was 4.23 (IQR, 2.65–6.43) in hospitalized patients and 3.64 (IQR, 2.16–6.13) in outpatients. Among hospitalized patients, antibody levels were classified as low in 1 (0.6%), medium in 104 (57.8%), and high in 75 (41.7%) patients; the corresponding numbers among outpatients were 0 (0%), 62 (67.4%), and 30 (32.6%), respectively.

### 3.2. Convalescent Plasma Antibody Levels

The distribution of antibody levels among the 180 hospitalized patients is presented in Table 2. Only one patient (0.6%) received CP with a low antibody level (<1.00), whereas 104 patients (57.8%) received CP with a medium antibody level (1.00–4.99) and 75 patients (41.7%) received CP with a high antibody level (≥5.00).

The median ELISA index was 4.23 (IQR, 2.65–6.43) among hospitalized patients and 3.64 (IQR, 2.16–6.13) among outpatients. The difference was not statistically significant (Mann–Whitney U test, *p* = 0.308) (Table 3).

Among hospitalized patients, the median ELISA index was 4.36 (IQR, 2.88–6.06; range, 1.10–84.75) in survivors and 4.16 (IQR, 2.32–6.65; range, 0.16–141.40) in non-survivors. The distributions did not differ significantly (Mann–Whitney U test, *p* = 0.427) (Table 4).

Analysis according to the study-specific antibody-level categories showed 30-day survival rates of 59.6% (62/104) in the medium-level group and 62.7% (47/75) in the high-level group. The corresponding odds ratio for 30-day mortality was 0.88 (95% CI, 0.48–1.62; Fisher’s exact *p* = 0.757) for the high- versus medium-level group. The single patient in the low-level category died within 30 days; because this category contained only one patient, it was excluded from inferential category comparisons (Table 5).

### 3.3. Timing of Convalescent Plasma Administration and Clinical Outcomes

Patients were classified according to the interval between symptom onset and CP administration as early (≤7 days) or late (>7 days) treatment. The combined associations of treatment timing and antibody levels with clinical outcomes are presented in Table 6.

Percentages were calculated within each treatment-timing and antibody-level stratum. Among hospitalized patients, 30-day clinical status was recorded in mutually exclusive categories of death, ongoing ICU care, or discharge. The early-treatment group comprised 119 patients (65 medium and 54 high antibody levels), whereas the late-treatment group comprised 61 patients (1 low, 39 medium, and 21 high antibody levels).

Among hospitalized patients who received CP within seven days of symptom onset, 30-day mortality was 37.0% (20/54) among those receiving plasma with high antibody levels and 38.5% (25/65) among those receiving plasma with medium antibody levels. Ongoing ICU care at day 30 was recorded in 3.7% (2/54) and 9.2% (6/65) of patients, respectively.

Among hospitalized patients who received CP more than seven days after symptom onset, 30-day mortality was 38.1% (8/21) among those receiving plasma with high antibody levels and 43.6% (17/39) among those receiving plasma with medium antibody levels. Ongoing ICU care at day 30 was recorded in 4.8% (1/21) and 5.1% (2/39) of patients, respectively.

Mortality was numerically lower in the high-antibody-level group than in the medium-antibody-level group in both timing strata; however, neither comparison was statistically significant (≤7 days: OR, 0.94; 95% CI, 0.45–1.98; *p* = 0.873; >7 days: OR, 0.80; 95% CI, 0.27–2.36; *p* = 0.681). ICU-care proportions were also not significantly different between the high- and medium-level groups (≤7 days: Fisher’s exact *p* = 0.290; >7 days: Fisher’s exact *p* = 1.000). These subgroup comparisons were considered exploratory because of the small cell sizes.

When the ELISA index was evaluated continuously after log2 transformation among hospitalized patients, it was not associated with 30-day mortality (OR per doubling, 0.89; 95% CI, 0.67–1.19; *p* = 0.425). The untransformed ELISA distributions were also similar between survivors and non-survivors (Mann–Whitney U *p* = 0.427).

The interval from symptom onset to CP administration was not associated with mortality when analyzed continuously (OR per day, 1.05; 95% CI, 0.98–1.12; *p* = 0.132). The formal early-treatment-by-log2-ELISA interaction was not statistically significant (interaction OR, 1.90; 95% CI, 0.94–3.84; *p* = 0.074).

In the exploratory complete-case multivariable model (*n* = 168; 66 deaths), the log2-transformed ELISA index remained unassociated with 30-day mortality after adjustment for available covariates (adjusted OR per doubling, 0.85; 95% CI, 0.51–1.42; *p* = 0.544). The high-versus-medium study-specific category comparison was likewise non-significant (OR, 0.88; 95% CI, 0.48–1.62; Fisher’s exact *p* = 0.757). These estimates were considered exploratory because of retrospective covariate ascertainment and residual confounding.

## 4. Discussion

This retrospective study examined associations between CP-related characteristics and outcomes within a treated cohort. Hospitalized patients had higher 30-day mortality than patients treated initially as outpatients; this difference should primarily be interpreted as reflecting major baseline differences in disease severity and treatment indication. ELISA antibody level was not associated with mortality in either categorical or continuous analyses. Although crude subgroup percentages differed by treatment timing and antibody category, confidence intervals were wide, and the formal interaction analysis was not statistically significant. The findings therefore do not demonstrate CP effectiveness or treatment-effect modification.

During the early phase of the COVID-19 pandemic, CP therapy was considered one of the most promising passive immunotherapy strategies because effective antiviral agents and vaccines were not yet widely available. Early observational studies suggested that administration of high-antibody-level CP, particularly during the early stage of infection, could reduce mortality and accelerate clinical recovery [7,8,9]. However, subsequent randomized controlled trials, including the RECOVERY trial and the study by Simonovich et al., failed to demonstrate a significant mortality benefit among hospitalized patients with COVID-19 [10,11]. Likewise, systematic reviews and meta-analyses concluded that the available evidence did not support the routine use of CP in the overall patient population, emphasizing substantial heterogeneity regarding patient selection, treatment timing, neutralizing antibody levels, and concomitant therapies [12,13,14].

Patients treated initially as outpatients had substantially higher 30-day survival than hospitalized patients. This comparison is not an estimate of CP benefit: the two groups received CP for different clinical indications and differed in baseline severity and comorbidity burden. The result is compatible with confounding by indication and the natural prognostic difference between clinically stable high-risk outpatients and patients already requiring hospitalization.

Within hospitalized patients, the continuous ELISA index was not associated with 30-day mortality. Comparisons of the medium and high study-specific categories likewise produced imprecise estimates, and the single patient in the low category precluded meaningful inference for that stratum. Earlier reports have suggested that early antibody-rich CP may be more useful in selected populations [15,16,17,18,19], but the present observational data neither confirm nor refute a therapeutic effect because there was no untreated comparator and subgroup cell sizes were limited.

The timing-by-ELISA interaction did not reach statistical significance. Accordingly, the observed subgroup percentages should not be described as a consistent beneficial trend. They are best regarded as hypothesis-generating observations that require confirmation in adequately powered studies with prespecified exposure thresholds, standardized neutralization assays, detailed baseline severity measures, and an appropriate comparator group.

Most patients were treated during the early pandemic period, when variants, vaccination coverage, and concomitant COVID-19 therapies changed over time. Calendar period and therapies such as corticosteroids, antivirals, anticoagulation, tocilizumab, and other immunomodulatory treatments could not be fully standardized and may have confounded the observed associations.

The study’s strengths include a clearly defined single-unit exposure, maintenance of the initial treatment-setting cohorts, and simultaneous evaluation of timing and ELISA antibody levels. Nevertheless, these features do not overcome the limitations inherent in retrospective comparisons between clinically different patient groups.

This study has important limitations. It was retrospective, single-center, and lacked a standard-care or no-CP comparator, preventing causal inference regarding CP effectiveness. Treatment indication, baseline severity, oxygen requirement, product availability, concomitant therapy, and calendar period may have influenced both exposure and outcome. Symptom onset was retrospectively ascertained and may have been misclassified. Neutralizing activity and recipient serostatus were unavailable. The low-antibody category contained one hospitalized patient, several subgroup cells were small, and the interaction analysis was underpowered. Missing covariate data required complete-case regression, and residual confounding remains likely despite adjustment.

## 5. Conclusions

In this retrospective cohort of CP-treated patients, the ELISA antibody level of the transfused unit was not independently associated with 30-day mortality. The timing-by-antibody interaction was also not statistically significant. These exploratory findings describe associations among treated patients and should not be interpreted as evidence that CP was effective or that one timing or antibody category was superior. Future studies of passive immunotherapy should use validated functional antibody assays, standardized exposure definitions, detailed severity adjustment, and an appropriate comparator group.

## Figures and Tables

**Table 1:** Demographic and clinical characteristics of patients according to the initial treatment setting.

Characteristic	Hospitalized (*n* = 180)	Outpatient (*n* = 92)	*p* Value
**Demographic characteristics**			
Age, years, mean ± SD	68.6 ± 13.8	66.3 ± 9.5	0.117
Female sex, *n* (%)	91 (50.6)	48 (52.2)	0.801
**Comorbidities, *n* (%)**			
Hypertension	132 (73.3)	47 (51.1)	0.0001
Diabetes mellitus	69 (38.3)	23 (25.0)	0.028
Cardiovascular disease	55 (30.6)	16 (17.4)	0.019
Chronic lung disease	53 (29.4)	11 (12.0)	0.001
Chronic kidney disease	21 (11.7)	5 (5.4)	0.098
**CP-related characteristics**			
Time from symptom onset to CP, days, median (IQR)	5.5 (3–8)	4 (4–6)	0.002
Early CP administration (≤7 days), *n* (%)	119 (66.1)	92 (100.0)	0.0001
ELISA index, median (IQR)	4.23 (2.65–6.43)	3.64 (2.16–6.13)	0.308
**Antibody-level category, *n* (%)**			—
Low (<1.00)	1 (0.6)	0 (0.0)	
Medium (1.00–4.99)	104 (57.8)	62 (67.4)	
High (≥5.00)	75 (41.7)	30 (32.6)	

**Table 2:** Distribution of convalescent plasma antibody level categories among hospitalized patients (*n* = 180).

Antibody Level Category	*n*	%
Low (0.00–0.99)	1	0.6
Medium (1.00–4.99)	104	57.8
High (≥5.00)	75	41.7
**Total**	**180**	**100.0**

**Table 3:** Plasma ELISA indices according to initial treatment setting.

Group	*n*	Median ELISA Index (IQR)	Range	*p* Value
Hospitalized patients	180	4.23 (2.65–6.43)	0.16–141.40	0.308
Outpatients	92	3.64 (2.16–6.13)	1.11–172.90	—

**Table 4:** Plasma ELISA indices according to 30-day mortality among hospitalized patients.

30-Day Outcome	*n*	Median ELISA Index (IQR)	Range	*p* Value
Survivors	109	4.36 (2.88–6.06)	1.10–84.75	0.427
Non-survivors	71	4.16 (2.32–6.65)	0.16–141.40	—

**Table 5:** Thirty-day survival according to study-specific plasma antibody-level categories.

Plasma Antibody Level	Non-Survivors, *n* (%)	Survivors, *n* (%)	Total
Low (<1.00)	1 (100.0)	0 (0.0)	1
Medium (1.00–4.99)	42 (40.4)	62 (59.6)	104
High (≥5.00)	28 (37.3)	47 (62.7)	75
Total	71 (39.4)	109 (60.6)	180

**Table 6:** Thirty-day clinical status according to timing of convalescent plasma administration and antibody levels among hospitalized patients (*n* = 180).

Clinical Outcome	Antibody Level	≤7 Days, *n* (%)	>7 Days, *n* (%)
Death	Low (<1.00)	0 (0.0)	1 (100.0)
	Medium (1.00–4.99)	25 (38.5)	17 (43.6)
	High (≥5.00)	20 (37.0)	8 (38.1)
Ongoing ICU care at day 30	Low (<1.00)	0 (0.0)	0 (0.0)
	Medium (1.00–4.99)	6 (9.2)	2 (5.1)
	High (≥5.00)	2 (3.7)	1 (4.8)
Discharged by day 30	Low (<1.00)	0 (0.0)	0 (0.0)
	Medium (1.00–4.99)	34 (52.3)	20 (51.3)
	High (≥5.00)	32 (59.3)	12 (57.1)

## Data Availability

The data supporting this study’s findings are available upon request from the corresponding author.

## Abbreviations

The following abbreviations are used in this manuscript:

ARDS	Acute respiratory distress syndrome
CP	Convalescent plasma
COVID-19	Coronavirus disease 2019
CRP	C-reactive protein
ICU	Intensive care unit
MERS	Middle East respiratory syndrome
SARS-CoV-2	Severe acute respiratory syndrome coronavirus 2

## References

[1] Huang C., Wang Y., Li X., Ren L., Zhao J., Hu Y., Zhang L., Fan G., Xu J., Gu X. (2020). Clinical features of patients infected with 2019 novel coronavirus in Wuhan, China. Lancet.

[2] Chen N., Zhou M., Dong X., Qu J., Gong F., Han Y., Qiu Y., Wang J., Liu Y., Wei Y. (2020). Epidemiological and clinical characteristics of 99 cases of 2019 novel coronavirus pneumonia in Wuhan, China: A descriptive study. Lancet.

[3] Channappanavar R., Perlman S. (2017). Pathogenic human coronavirus infections: Causes and consequences of cytokine storm and immunopathology. Semin. Immunopathol..

[4] Marano G., Vaglio S., Pupella S., Facco G., Catalano L., Liumbruno G.M., Grazzini G. (2016). Convalescent plasma: New evidence for an old therapeutic tool. Blood Transfus..

[5] Li L., Zhang W., Hu Y., Tong X., Zheng S., Yang J., Kong Y., Ren L., Wei Q., Mei H. (2020). Effect of convalescent plasma therapy on time to clinical improvement in patients with severe and life-threatening COVID-19: A randomized clinical trial. JAMA.

[6] Kimber C., Lamikanra A.A., Geneen L.J., Sandercock J., Dorée C., Valk S.J., Estcourt L.J. (2023). A systematic review of the safety and efficacy of convalescent plasma or immunoglobulin treatment for people with severe respiratory viral infections due to coronaviruses or influenza. Transfus. Med..

[7] Salazar E., Christensen P.A., Graviss E.A., Nguyen D.T., Castillo B., Chen J., Lopez B.V., Eagar T.N., Yi X., Zhao P. (2020). Treatment of coronavirus disease 2019 patients with convalescent plasma reveals a signal of significantly decreased mortality. Am. J. Pathol..

[8] Libster R., Pérez Marc G., Wappner D., Coviello S., Bianchi A., Braem V., Esteban I., Caballero M.T., Wood C., Berrueta M. (2021). Early high-antibody-level plasma therapy to prevent severe COVID-19 in older adults. N. Engl. J. Med..

[9] Joyner M.J., Carter R.E., Senefeld J.W., Klassen S.A., Mills J.R., Johnson P.W., Theel E.S., Wiggins C.C., Bruno K.A., Klompas A.M. (2021). Convalescent plasma antibody levels and the risk of death from COVID-19. N. Engl. J. Med..

[10] Abani O., Abbas A., Abbas F., Abbas M., Abbasi S., Abbass H., Abbott A., Abdallah N., Abdelaziz A., Abdelfattah M. (2021). Convalescent plasma in patients admitted to hospital with COVID-19 (RECOVERY): A randomised, controlled, open-label, platform trial. Lancet.

[11] Simonovich V.A., Burgos Pratx L.D., Scibona P., Beruto M.V., Vallone M.G., Vázquez C., Savoy N., Giunta D.H., Pérez L.G., Sánchez M.d.L. (2021). A randomized trial of convalescent plasma in COVID-19 severe pneumonia. N. Engl. J. Med..

[12] Klassen S.A., Senefeld J.W., Johnson P.W., Carter R.E., Wiggins C.C., Shoham S., Grossman B.J., Henderson J.P., Musser J., Salazar E. (2021). The effect of convalescent plasma therapy on mortality among patients with COVID-19: A systematic review and meta-analysis. Mayo Clin. Proc..

[13] Ling R.R., Sim J.J.L., Tan F.L., Tai B.C., Syn N., Mucheli S.S., Fan B.E., Mitra S., Ramanathan K. (2022). Convalescent plasma for patients hospitalized with coronavirus disease 2019: A meta-analysis with trial sequential analysis of randomized controlled trials. Transfus. Med. Rev..

[14] Piechotta V., Iannizzi C., Chai K.L., Valk S.J., Kimber C., Dorando E., Monsef I., Wood E.M., Lamikanra A.A., Roberts D.J. (2021). Convalescent plasma or hyperimmune immunoglobulin for people with COVID-19: A living systematic review. Cochrane Database Syst. Rev..

[15] Casadevall A., Joyner M.J., Pirofski L.A., Senefeld J.W., Shoham S., Sullivan D., Paneth N., Focosi D. (2023). Convalescent plasma therapy in COVID-19: Unravelling the data using the principles of antibody therapy. Expert Rev. Respir. Med..

[16] Franchini M., Focosi D. (2023). The role of convalescent plasma in COVID-19: A conclusive post-pandemic review. Life.

[17] Filippatos C., Ntanasis-Stathopoulos I., Sekeri K., Ntanasis-Stathopoulos A., Gavriatopoulou M., Psaltopoulou T., Dounias G., Sergentanis T.N., Terpos E. (2023). Convalescent plasma therapy for COVID-19: A systematic review and meta-analysis of randomized controlled trials. Viruses.

[18] Senefeld J.W., Franchini M., Mengoli C., Cruciani M., Zani M., Gorman E.K., Focosi D., Casadevall A., Joyner M.J. (2023). COVID-19 convalescent plasma for the treatment of immunocompromised patients: A systematic review and meta-analysis. JAMA Netw. Open.

[19] Franchini M., Cruciani M., Casadevall A., Joyner M.J., Senefeld J.W., Sullivan D.J., Zani M., Focosi D. (2024). Safety of COVID-19 convalescent plasma: A definitive systematic review and meta-analysis of randomized controlled trials. Transfusion.

